# A Different Approach for Protruding Earlobe Correction—Modified Fish-tail Technique

**DOI:** 10.1007/s00266-024-03912-7

**Published:** 2024-03-04

**Authors:** Batuhan Sert, Meltem Ayhan Oral, Ozgur Turan, Ilker Uyar

**Affiliations:** 1 Department of Plastic, Reconstructive and Aesthetic Surgery, Izmir Katip Celebi University Medical Faculty, Izmir, Turkey; 2 Department of Plastic, Reconstructive and Aesthetic Surgery, Izmir Ataturk Training and Research Hospital, Izmir, Turkey

**Keywords:** Lobuloplasty, Protruded lobule, Otoplasty, Modified fish tail

## Abstract

**Background:**

Protruding ears are the most common auricular abnormalities seen in children (1). Protruding ears are a condition that has social and psychological consequences due to its physical appearance and one of the main causes of peer bullying at young ages (2). While various surgical methods exist to address prominent ears, the options for correcting the lobule are relatively scarce. In this study, we are aimed to present the modified fish-tail technique that we have developed and to compare it with other techniques in the literature.

**Methods:**

The patients were selected from the cases that underwent otoplasty for prominent ear correction in our clinic between 2020 and 2022. A total of 21 cases that required protruded lobule correction during otoplasty were included in our study. Keloid and hypertrophic scar formation, wound dehiscence, hematoma, infection and recurrence in the lobule were evaluated. The patients were followed up for at least 1 year for early and late complications.

**Results:**

Each patient in the study underwent bilateral prominent ear correction, including bilateral modified fish-tail technique. All cases were followed for at least 12 months. There was no wound dehiscence, infection, recurrence in lobule prominence or hematoma during the follow-up period. No hypertrophic scar or keloid was observed in any case.

**Conclusions:**

Our method stands out for its ability to achieve both adjustable vertical height and effective lobule correction with a reduced need for skin excision. We recommend the modified fish-tail technique as an alternative technique for prominent lobule surgery.

**Level of Evidence IV:**

This journal requires that authors assign a level of evidence to each article. For a full description of these evidence-based medicine ratings, please refer to the Table of contents or the online Instructions to Authors www.springer.com/00266.

## Introduction

Protruding ears are the most common auricular abnormalities seen in children. Etiologically, it is a hereditary malformation with familial accumulation, additionally environmental influences during the development and migration of the auricle in the second trimester represent another causative factor [1].

The reason for the increased projection may be a lack of rotation of the cavum with an enlargement of the cavum–mastoid angle. This causes to protrusion of the auricle and pseudoconcha hyperplasia. Similarly, the less common real concha hyperplasia can result in increased projection of auricle, additionally antihelix hypoplasia is very common in protruding ears. The position of the lobule depends on the cavum rotation and the antihelix fold. However, it can also be stuck out or be malformed regardless of the remaining shape of the ear [1].

Protruding ears are a condition that has social and psychological consequences due to its physical appearance and one of the main causes of peer bullying at young ages. This situation causes psychological and traumatic damage as a result of the public's increased exposure to social media [2].

Various surgical methods exist to address prominent ears. Prominent ear surgery is an operation with very effective results under local or general anesthesia. In this procedure, mustarde sutures are used to redesign the antihelix and furnas sutures are used for concho-scaphal fixation [3, 4]. However, the options for correcting the lobule are relatively limited. Even after correcting the auricle in this operation, telephone ear or reverse telephone ear deformity may be encountered if lobuloplasty is not included in the procedure. [5]

In this study, we are aimed to present the modified fish-tail technique that we have developed and to compare it with other techniques in the literature.

## Materials and Methods

The study was approved by the local ethics committee. The patients were selected from the cases that underwent otoplasty for prominent ear correction in our clinic between 2020 and 2022. A total of 21 cases that required protruded lobule correction during otoplasty were included in our study. Patients with comorbidities, previous prominent ear surgery and patients who did not attend regular check-ups were excluded from the study. All cases were examined before surgery, patient expectations were noted and the skin excision pattern was drawn in the preoperative preparation room, and then the operation was planned. All cases were operated by the authors under local or general anesthesia. Keloid and hypertrophic scar formation, wound dehiscence, hematoma, infection and recurrence in the lobule were evaluated. The patients were followed up for at least 1 year for early and late complications.

### Surgical Technique

Depending on the same otoplasty technique in all cases, the operation was started after general or local anesthesia was applied and the preparation was completed. Postauricular excess skin was excised in an elliptical shape. Subsequently, excess conchal cartilage was marked with methylene blue, excised as needed and the cartilage edges were sutured together. Following this, as described in the technique of the transcutaneous fixation-assisted method, temporary mattress sutures were placed on the front of the ear from the antihelix medial to the antihelix lateral with 3.0 round silk suture material to define the antihelix fold. The concha and scapha cartilages that came close to each other at the back of the ear were fixed together with mustarde sutures [6]. Subsequent to this, furnas sutures were applied to narrow the concha–mastoid angle. Following the completion of the otoplasty technique, lobule prominence was re-evaluated.

**Fig. 1:**
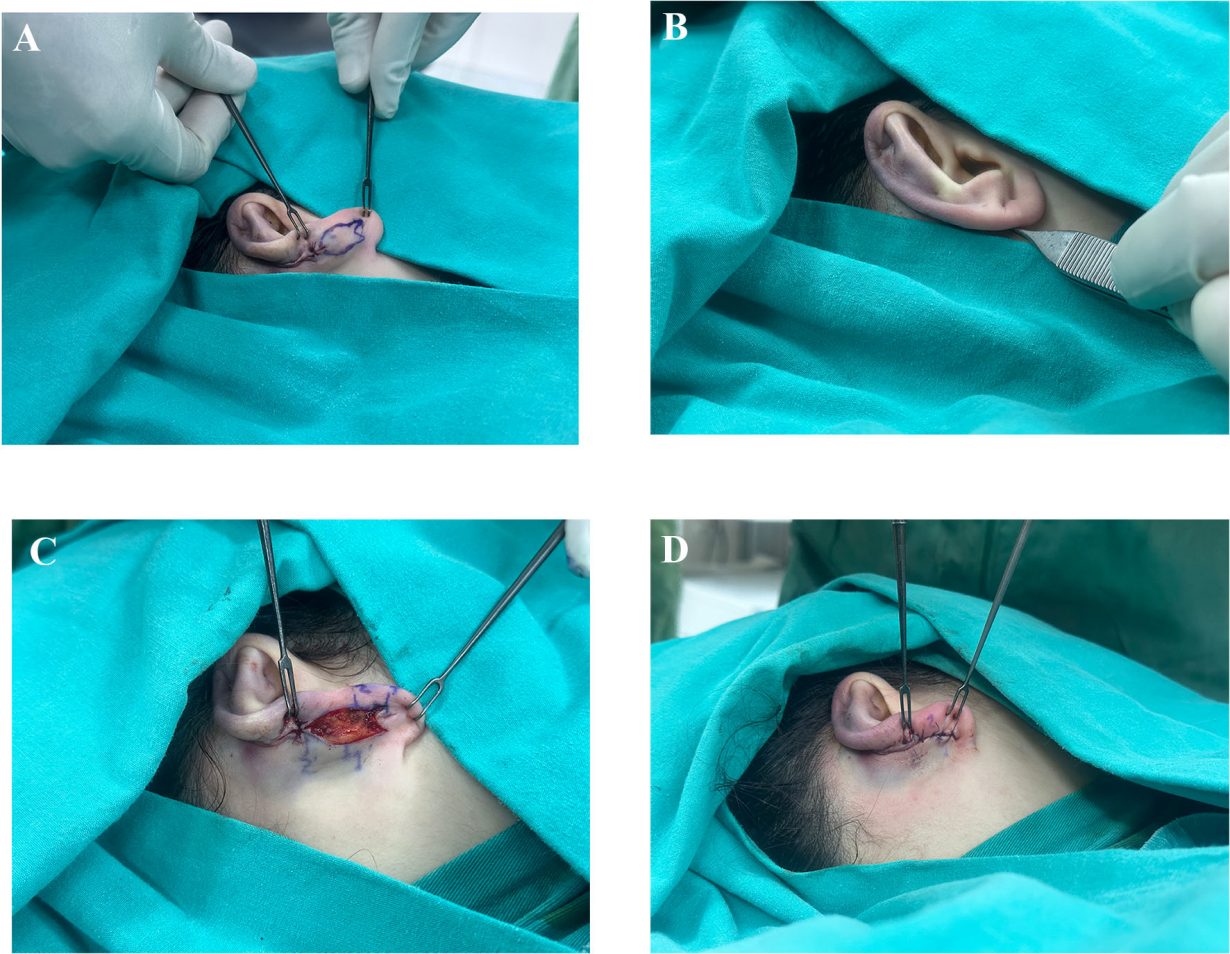
**A:** Modified fish-tail excision pattern determined after classic otoplasty surgery**. B:** The skin at the most inferolateral side of the excision border was pulled with forceps toward the skin on the superomedial side of the excision border, and the most accurate suturing point for lobule correction was determined**. C** Following this, the points to be sutured together were determined with methylene blue, point 1 was sutured to point 1’ and point 2 was sutured to point 2’.** D** Asymmetric oblique suturation was applied to suture gaps

Vertically extending fish-tail excision borders were marked with methylene blue on the posterior lobule region, starting from the inferior end of the preceding elliptical excision. (Fig. 1A) An important point here is that maintaining a limited horizontal width for the fish-tail excision borders is necessary to prevent the appearance of a narrowed and bulky lobule when observed from a lateral angle. Additionally, the incision must be completed at least 0,5 cm above the lowest border of the lobule to ensure the scar remains discreet. After that, the fish-tail excision was completed, and during suturation, the skin at the most inferolateral side of the excision border was pulled with forceps toward the skin on the superomedial side of the excision border, the most accurate suturing point for lobule correction was determined (Fig. 1B). Following this, the points to be sutured together were determined with methylene blue, (Fig. 1C) point 1 was sutured to point 1’ and point 2 was sutured to point 2’. Following this, asymmetric oblique suturation was applied to suture gaps (Fig. 1D). If needed, the triangular region at the most inferior of the excision margin can be sutured by pulling it superiorly in order to move the inferior part of the lobule to the superoposterior, or it can be directly excised for dog-ear correction. Additionally, it can ensure more permanence if the inferolateral points obliquely suspend to the conchal cartilage. Afterward, all remaining incisions were repaired using continuous matress suturation technique with 3-0 monocryl (Ethicon, USA) suture.

## Results

**Fig. 2:**
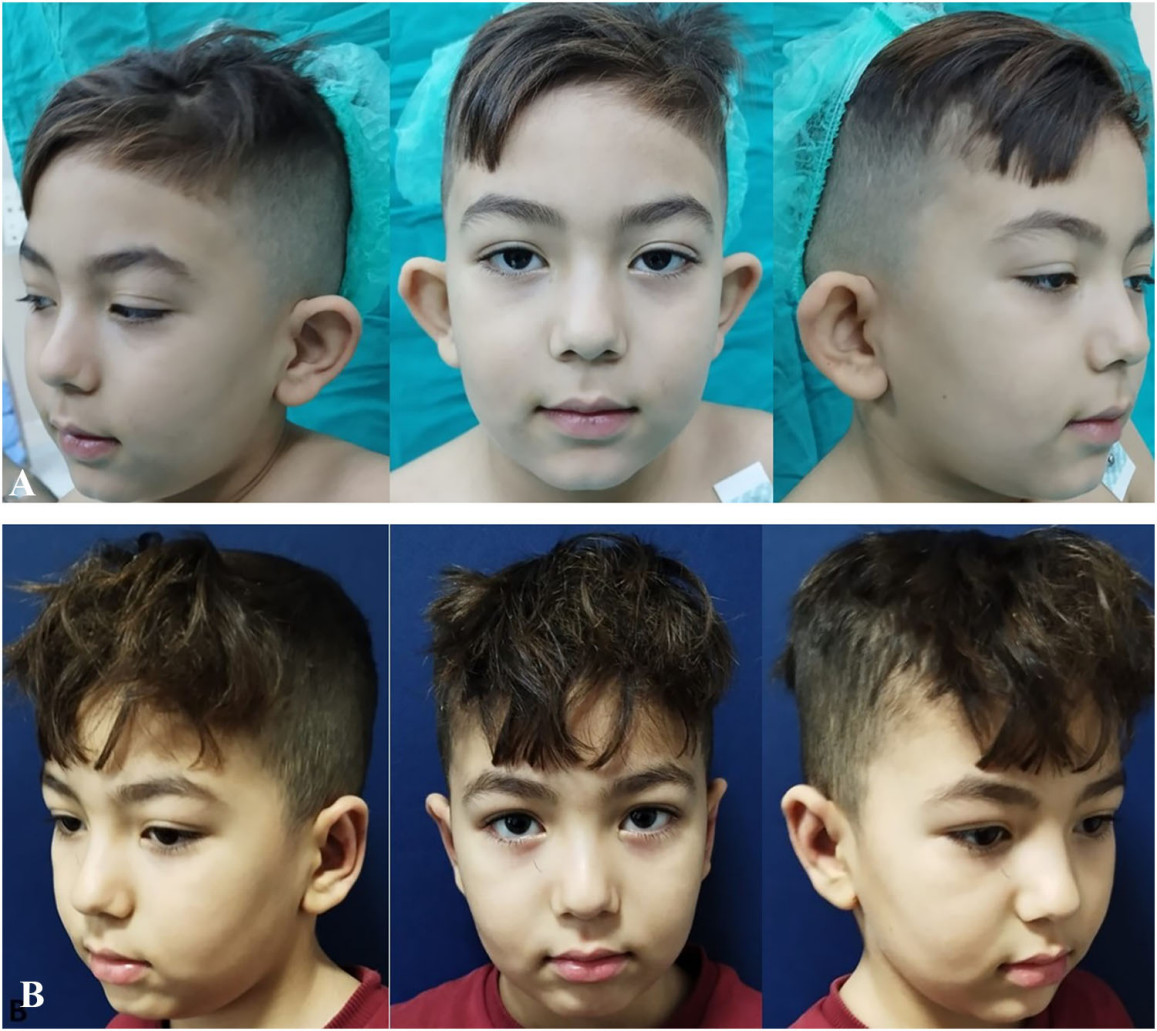
**A:** Preoperative. B Postoperative first year

**Fig. 3:**
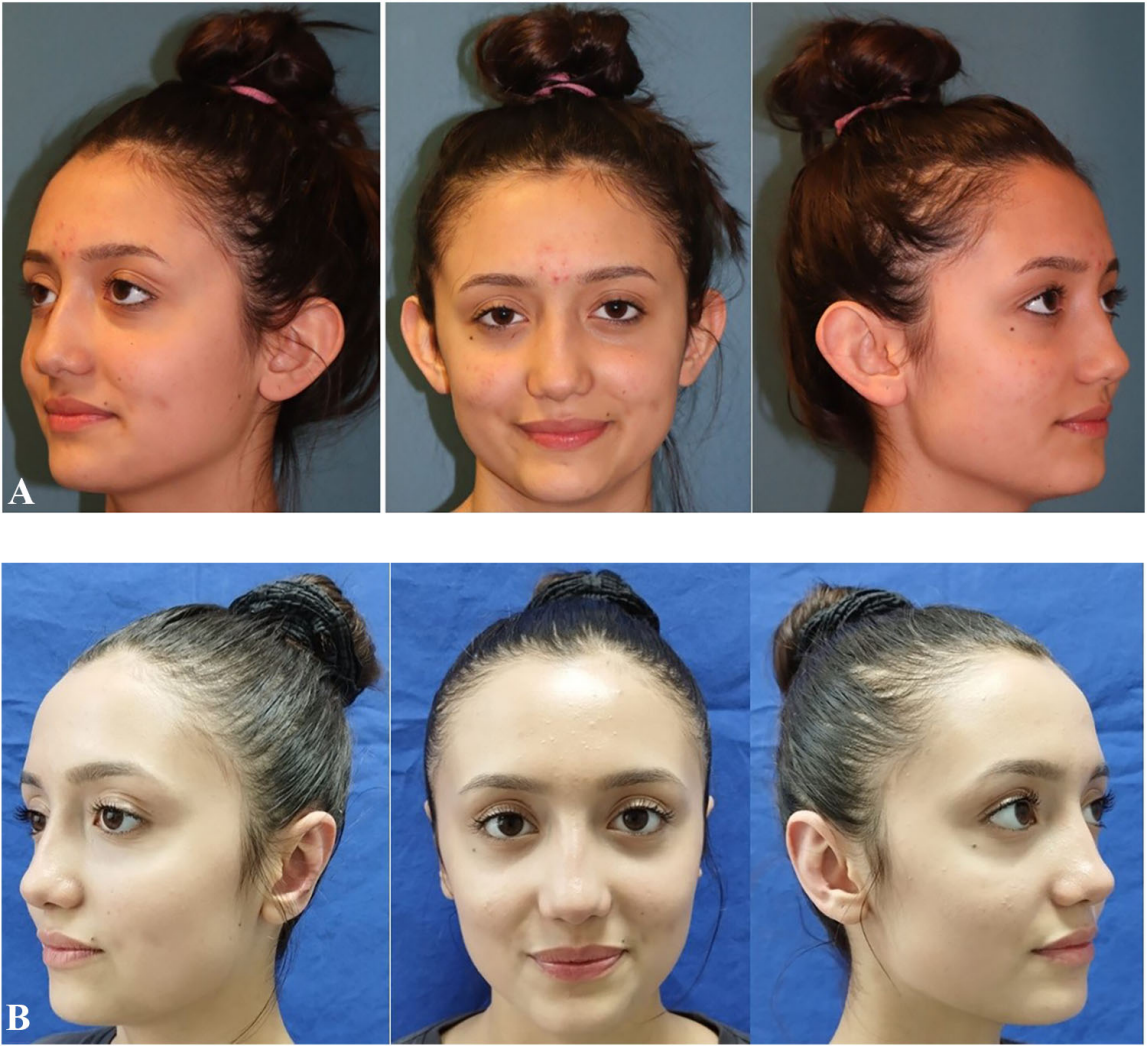
**A**: Preoperative. **B**: Postoperative first year

**Fig. 4:**
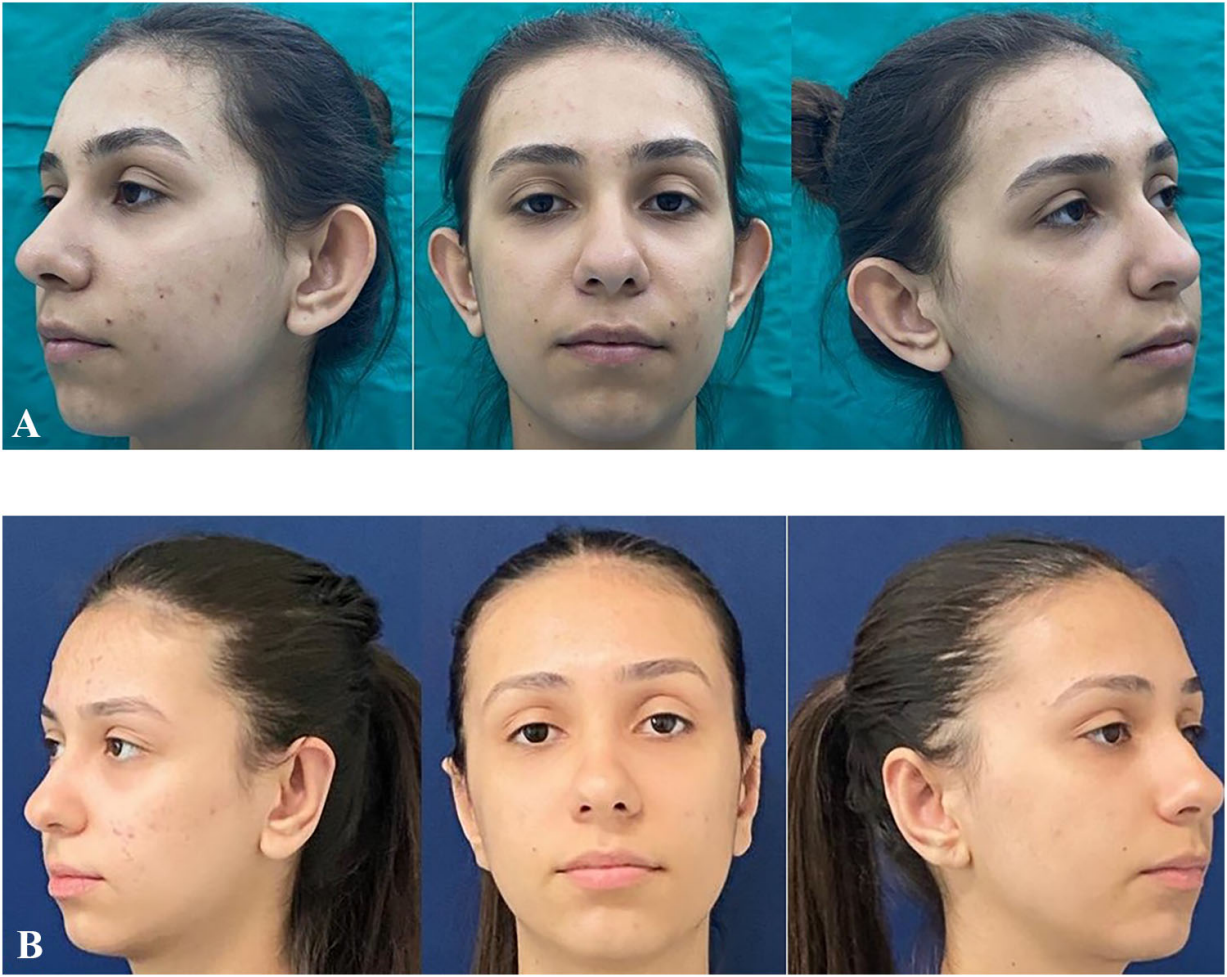
**A**: Preoperative. **B**: Postoperative first year

The average age of the patients was 14 years (5–17). Among the 21 patients, 14 (66%) were female and seven (33%) were male. All patients underwent bilateral prominent ear correction and bilateral modified fish-tail excision. All cases were followed for at least 12 months. The average follow-up period was 15 months (range, 12–24) (Table 1). The scars exhibited a typical appearance and were well hidden in the postauricular region. Throughout the follow-up, there were no instances of wound dehiscence, infection, recurrence in lobule prominence or hematoma. Furthermore, no hypertrophic scars or keloids were observed in any case. A thorough comparison of preoperative, early and late postoperative photographs was conducted for each case. (Figs. 2, 3 and 4).

**Table 1:** Demographic data of the patients

Patients	Age	Sex	Follow-up Period	Type of Anesthesia
1	5	Male	18 months	General anesthesia
2	15	Female	12 months	Local Anesthesia
3	15	Female	15 months	Local Anesthesia
4	12	Female	24 months	Local Anesthesia
5	14	Female	15 months	Local Anesthesia
6	14	Female	15 months	Local Anesthesia
7	17	Female	12 months	Local Anesthesia
8	14	Female	18 months	Local Anesthesia
9	17	Male	24 months	Local Anesthesia
10	15	Female	18 months	Local Anesthesia
11	7	Female	18 months	General Anesthesia
12	17	Female	24 months	Local Anesthesia
13	16	Male	12 months	Local Anesthesia
14	14	Female	12 months	Local Anesthesia
15	17	Male	12 months	Local Anesthesia
16	17	Male	15 months	Local Anesthesia
17	10	Female	12 months	General Anesthesia
18	16	Female	15 months	Local Anesthesia
19	16	Male	12 months	Local Anesthesia
20	15	Female	12 months	Local Anesthesia
21	17	Male	15 months	Local Anesthesia

## Discussion

The most common indication for otoplasty is the correction of ears with increased prominence but normal structure. Protruding ears are a condition that has psychological and social consequences due to its physical appearance. The correction of protruding ears through otoplasty often results in high satisfaction rates for both the patient and their family [2]. Despite the abundance of the literature discussing the objectives and techniques of otoplasty surgery, there is a noticeable scarcity of articles specifically addressing lobuloplasty techniques.

Prominent ear surgery, like any surgical procedure, can be associated with both early and late complications. The most common early complication is hematoma. Additionally, bleeding, skin necrosis, dehiscence and infection are other early complications. In the late period, complications such as hypertrophic scars, keloid scars, hypersensitivity, suture exposure, telephone ear deformity, inadequate or overcorrection and recurrence may occur. Facial paralysis, though exceptionally rare, is also reported in the literature [7, 8]. Remarkably, no complications were encountered in our study.

Following the application of classical otoplasty techniques, lobule prominence may appear increased. Therefore, lobuloplasty should be considered as the last stage of otoplasty. The aim of lobuloplasty is to align the helical rim and ear lobule on the same straight line [9]. This strategic approach helps prevent deformities arising from overcorrection or undercorrection, such as the occurrence of telephone ear or reverse telephone ear.

Gosain suggested a single-suture approach in which the helical tail is fixed to the mastoid region to correct the position of the lobule [5]. Single-suture approach may be simple and durable, but the mastoid fascia that the suture passes is fairly vascular and may cause bleeding.

Spira et al. advocated an approach that a suture fixed to the periosteum between the dermis and the scalp following wedge excision [10]. This approach may provide long-term durability, but may cause complications such as hematoma due to the necessary dissection of the mastoid region.

Numerous authors attribute lobule prominence to excess skin. They look for a solution in excision techniques aimed at removing this surplus skin. Some of these have suggested fat resection in addition to skin excision in the lobule area with a Z-plasty or an ellipse [9].

**Fig. 5:**
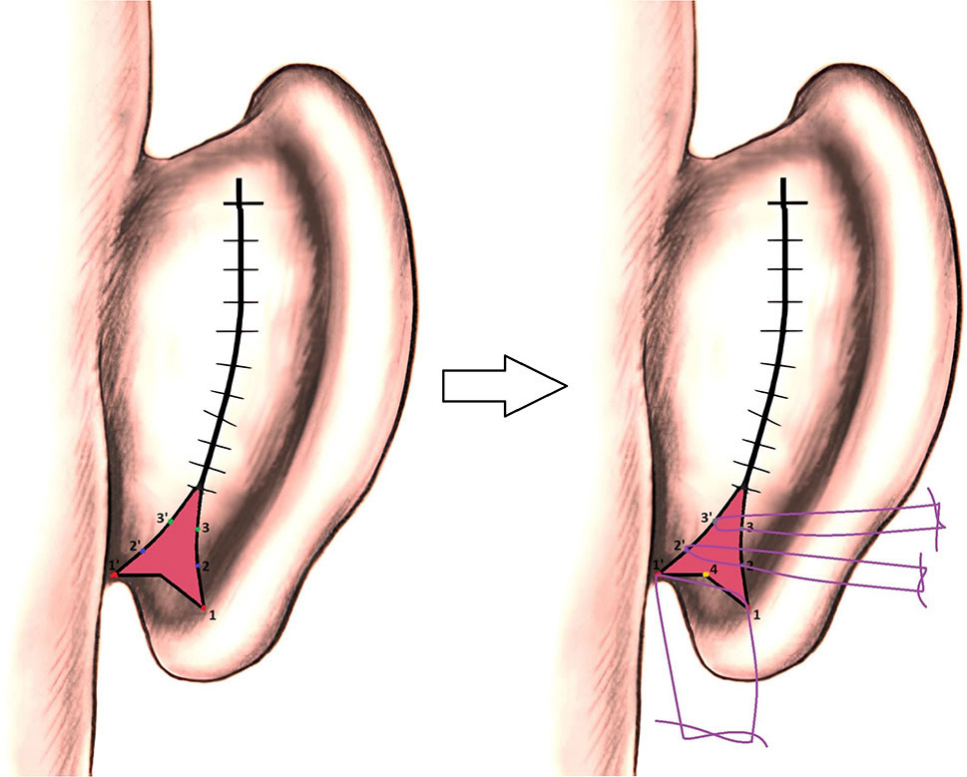
Wood-Smith suggested a “fish-tail-like” retrolobular skin excision followed by V–Y plasty**.** This technique allows for the adjustment of the vertical height of the lobule as desired (with suturing “point 4” to a higher point), offering potential permanence by suturing the superior part of the helical tail (point 4) to the conchal cartilage.

Wood-Smith suggested a “fish-tail-like” retrolobular skin excision followed by V–Y plasty (11) (Fig. 5). This technique allows for the adjustment of the vertical height of the lobule as desired, offering potential permanence by suturing the superior part of the helical tail to the conchal cartilage. However, it comes with the drawback of requiring a broad skin excision for adequate correction. Accordingly, it has disadvantages such as the anterior part of the lobule folding over during suturing, resulting in a narrower, bulkier lobule and a visible tight suture line. Additionally, there is a predisposition to keloid and hypertrophic scars with this method.

**Fig. 6:**
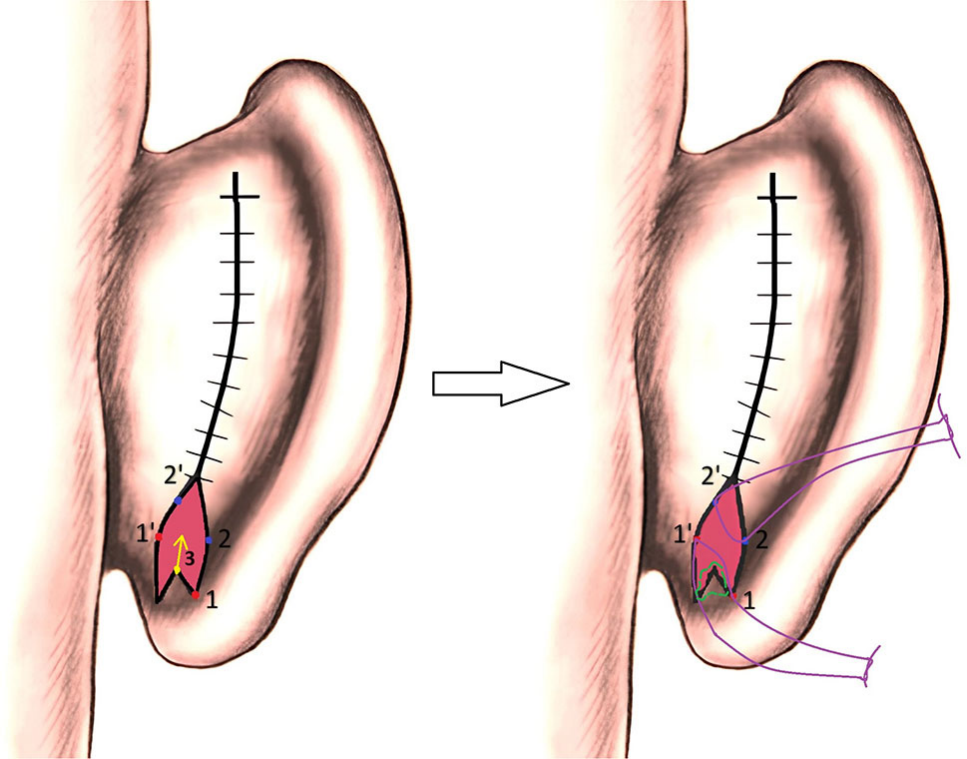
Our modified fish-tail technique offers several advantages, including the ability to achieve adjustable vertical height (Point 3 and yellow arrow) and sufficient correction through asymmetric oblique suturation (purple lines), all with minimal skin excision. Additionally, if vertical height is sufficient, the triangular region at the most inferior of the excision margin can be directly excised for dog-ear correction. (green lines)

Our modified fish-tail technique offers several advantages, including the ability to achieve adjustable vertical height and sufficient correction through asymmetric oblique suturation, all with minimal skin excision (Fig. 6). An essential feature is the adaptability of the technique; if the initial oblique sutured point does not yield sufficient correction, the sutures can be reopened and adjusted to a higher point without the need for additional excision. By obliquely suspending the inferolateral points to the conchal cartilage, our method enhances the likelihood of long-term success. Moreover, the technique is designed to reduce the potential for keloid-hypertrophic scar development, thanks to the obliquely placed sutures and reduced tension. Additionally that no recurrence was observed in the 1-year follow-up of the cases and the scar was hidden in the postauricular region.

We emphasize the retrospective nature of the study, the short follow-up period and the small number of cases included as study limitations.

## Conclusion

Our approach enables adjustable vertical height and sufficient lobule correction with reduced skin excision. We believe that this technique minimizes the likelihood of developing keloid and hypertrophic scars due to the application of less tension during suturing. We recommend the modified fish-tail technique as an alternative method for prominent lobule surgery.
